# Alteration of Phenolic and Volatile Compounds of Tea Leaf Extract by Tyrosinase and *β*-Glucosidase during Preparation of Ready-to-Drink Tea on Farm

**DOI:** 10.1155/2022/1977762

**Published:** 2022-03-01

**Authors:** Dea Risfika Faustina, Rachmad Gunadi, Aprilia Fitriani, Supriyadi Supriyadi

**Affiliations:** ^1^Department of Food and Agricultural Product Technology, Faculty of Agricultural Technology, Universitas Gadjah Mada, Yogyakarta 55281, Indonesia; ^2^Department of Soil Science, Faculty of Agriculture, Universitas Gadjah Mada, Yogyakarta 55281, Indonesia; ^3^Food Technology, Faculty of Industrial Technology, Universitas Ahmad Dahlan, Jenderal Ahmad Yani Street, Banguntapan, Bantul, Yogyakarta, Indonesia

## Abstract

The manufacturing of ready-to-drink black tea was through a long process, up to 14-18 hours. There was an alternative way to produce RTD black tea directly on the farm to reduce time production and improve the quality of black tea using exogenous enzymes, i.e., tyrosinase and *β*-glucosidase. Tyrosinase was investigated for the ability to improve the color of tea extract by oxidize the phenolic content of green tea leaves to theaflavin and thearubigin, and *β*-glucosidase can enhance the volatile compounds by hydrolyze glycosidic bonds in tea leaves. Incubation of tea leaf extract with tyrosinase produces a high content of theaflavin and good color of tea extract but lowered the antioxidant activity. According to the TF/TR ratio values, tyrosinase treated tea leaf extract was in the best quality tea range. The use of *β*-glucosidase showed an increase in the proportion of good volatile compounds of linalool, linalool oxide, methyl salicylate, and *β*-damascenone.

## 1. Introduction

Tea is the second most consumed drink globally, after plain water (Rietveld, 2003). There are three types of tea: green tea (unfermented tea), oolong tea (semi-fermented tea), and black tea (full fermented tea). Black tea is the most consumed tea globally, up to 76-78% [1]. The manufacturing of black tea was through a long process, for orthodox black tea manufacturing, up to 14-18 hours [2]. The plucked tea leaves were withered, bruised, and rolled to extract the juice and through the oxidation process and dried [3]. The previous study had tried to reduced the processing time and improved the quality of black tea using exogenous enzymes. The use of polyphenol oxidase, i.e., 1000 U/g laccase on tea freshly cut rooibos leaves, could improve the color and aroma of rooibos tea and reduce the processing time from 14-16 hours to 2 hours [4]. Used of laccase and tyrosinase was compared. Fresh tea leaf extract treated with tyrosinase was produced the highest concentration of theaflavin at 40 minutes of incubation. After 60 minutes of incubation, theaflavin decreased, and after 120 minutes of incubation, as much as 70% theaflavin remained. Meanwhile, only 20% of theaflavin remained after 120 minutes of incubation of laccase because laccase can further react with theaflavin and produced less yellow color [5]. Tyrosinase, laccase, bilirubin oxidase, and crude polyphenol oxidase from *Camellia sinensis* were used as polyphenol oxidase to incubated with catechin. Among these polyphenol oxidases, tyrosinase showed as the best polyphenol oxidase to produced more than 80% of theaflavin, whereas laccase and bilirubin oxidase produce about 5% of theaflavin, and crude polyphenol oxidase from *Camellia sinensis* produces 27% of theaflavin [6]. Thus, tyrosinase was the best polyphenol oxidase to oxidized catechin, produced higher theaflavin content, and produced a better yellow color [5, 6]. The quality of the black tea depends on the aroma, color of the tea infusion, and the components in it [7]. Meanwhile, there is no study about the volatile compounds of the tyrosinase treated fresh tea leaf extract.

One of the precursors of the volatile compound was glycosides. These flavor precursors have a glycosidic bond that bonds a sugar moiety to a functional group and aromatic parts. Glycoside that has been isolated in the fresh tea leaves was *β*-D-glucopyranosides [8], *β*-primeverosides, and *β*-vicianosides with aglycons of linalool, linalool oxides, geraniol, benzyl alcohol, and methyl salicylate [9]. *β*-Glucosidase is a glycoside hydrolase type enzyme that can hydrolyze glycosidic bonds in tea leaves. *β*-Glucosidase contributes to the formation and development of the floral tea aroma by releasing volatile compounds [10, 11]. Hot extraction during milky tea beverage processing loosed the aroma substance; therefore, *β*-glucosidase was added to improve the volatile aroma. The aroma substances generated from glycosidic bound proved to increase twice compared to the control [12]. There is the possibility to use a combination of polyphenol oxidase and glycoside hydrolase type enzyme to improve the color and aroma of tea extract.

Currently, there is another way to drink tea, i.e., consuming ready-to-drink (RTD) tea. The consumption of RTD tea was the highest compared to other RTD beverages [13]. RTD tea is ready-prepared black or green tea. RTD tea can be produced by diluting powder or concentrated tea extract or extracting tea leaves with hot water and filtered it to removed residual and insoluble particles [14]. The use of exogenous enzymes, i.e., tyrosinase and *β*-glucosidase, has a prospect as an alternative to RTD tea processing directly held on the farm. Therefore, this research was conducted to improve the brew color and aroma of black tea prepared from the tea leaf extract. This research will form the basis for developing the RTD industry directly on the farm to suppress the conventional RTD production process.

## 2. Methods

### 2.1. Materials

Fresh tea leaves (superior clone-clone of PGL 15) were picked after the ages of 5 years, and 25 days after, the shoot tip was grown. Fresh tea leaves (superior clone-clone of PGL 15) with shoot tips and two leaves underneath (*P* + 2) were obtained from Universitas Gadjah Mada tea plantation, namely, Pagilaran Plantation site, Batang, Central Java, Indonesia (1000 above sea level), which plucked in September 2020. Tyrosinase (7,164 U/mg solid) was purchased from Sigma-Aldrich. *β*-Glucosidase (293 U/mg solid) was purchased from Xi'an Geekee Biotech (China). Catechin standard was purchased from Sigma-Aldrich. Methanol and acetonitrile used were HPLC grade and or analytical grade quality. Broken Orange Pekoe (BOP) black tea produced by Pagilaran Plantation used a commercial black tea to be compared with the tyrosinase treatments.

### 2.2. Method

#### 2.2.1. Preparation of Tea Extract

The extraction method of tea leaves is based on the previous study [5] with modifications. As much as 8.3 grams of fresh hand-picked tea leaves (pekoe, two leaves, and a bud) was steamed using steamer above the boiled water for 3 minutes to deactive the enzymes then extracted with 100 mL of water at 85°C for 20 minutes and filtered using No. 1 Whatman paper then centrifuged at 23°C, 3000 x g for 30 minutes. Extraction was repeated three times.

#### 2.2.2. Application of Tyrosinase and *β*-Glucosidase on Tea Leaf Extract

Enzyme treatment on tea leaf extract was carried out according to the previous study [5] with modifications. As much as 1 mL of the tea extract was added with 1 mL tyrosinase solution (111 U/mL, 446 U/mL, and 1785 U/mL) and incubated at 25°C for 40 minutes. Then, the tea extract was heated at 55°C for 15 minutes to stop the enzymatic reaction. Then, the mixture was added with 7.4 U/mL as much as *β*-glucosidase 16 *μ*l and incubated at 50°C for 2 hours, then heated at 85°C for 15 minutes to stop the enzymatic reaction [12]. The application of tyrosinase and *β*-glucosidase on tea leaf extract was repeated three times for each treatment.

#### 2.2.3. Identification of Catechin Profile and Gallic Acid

Quantification of catechin and gallic acid was according to the previous study [15]. A total of 20 *μ*L of tea extract samples were first filtered with 0.45 *μ*m nylon filter membrane then automatically injected into the HPLC injector DAD Shimadzu Japan series equipped with a PDA detector. The column used was a 5 *μ*m C18 Shimadzu column (SHIMPACK GIST 4.6 × 150 mm), in an oven temperature of 40°C, detection wavelength of 210 nm, and running time of 35 minutes. A Shimadzu LC-20AB pump was operated in isocratic mode with a mobile phase containing a mixture of 0.1% orthophosphoric acid, water, acetonitrile, and methanol (14 : 7: 3: 1, v/v/v/v). The spectra range used 190–440 nm. The mobile phase flow rate was maintained at 1 mL/minute. Retention times and spectra were compared with those of pure standards within that range. The amounts of different catechin in the samples were determined by HPLC [16]. Calibration curves were constructed with catechin EC (epicatechin), ECG (epicatechin gallate), EGC (epigallocatechin), and EGCG (epigallocatechin gallate), and gallic acid compounds. The quantification of catechin compounds and gallic acid was carried out using external standard catechin epicatechin (EC), epicatechin gallate (ECG), epigallocatechin (EGC), epigallocatechin gallate (EGCG), and gallic acid compounds, respectively.

#### 2.2.4. Analysis of Theaflavin and Thearubigin

As much as 1 mL of tea extract was mixed with 0.3 mL of 2.5% (w/v) of aqueous sodium hydrogen carbonate solution and extracted with 1 mL ethyl acetate, then vortexed for 1 minute. Then, the upper layer named the ethyl acetate layer (TF fraction) was used in the analysis of TF. 1 mL of TF fraction was diluted to 2.5 mL with methanol and named extract solution 1 (E1). Extract solution 2 (E2) was mixed with 1 mL of tea extract, 1 mL of 10% (w/v) aqueous saturated oxalic acid, 8 mL of aquadest, and adjusted to 25 mL with methanol. Optical densities E1 and E2 at 380 nm at Spectrophotometer UV-Vis Thermoscientific GENESYS 150 were used to determine the content of TF and TR and calculated with the following equation [17]:

(1) $$\begin{array}{c} \text{TF }\left(\%\right)=2.25\text{ x E}1,\\ \text{TR }\left(\%\right)=7.06\text{ x }\left(4\text{E}2-\text{E}1\right). \end{array}$$

#### 2.2.5. Analysis of Total Phenolic Content

Analysis of total phenolic content was prepared by mixing a 0.5 mL of tea extracts with a 2.5 mL 10% Folin Ciocalteu reagent and 2 mL of 7.5% Na_2_CO_3_ solution. Then, the solution was vortexed and stored in a dark room for 1 hour, then its absorbance measured at a wavelength of 765 nm using Spectrophotometer UV-Vis Thermoscientific GENESYS 150. Total phenol content (mg GAE/g) was calculated based on the standard gallic acid curve. The standard solution is used in the form of a gallic acid solution concentrating 200 *μ*M – 500 *μ*M [18].

#### 2.2.6. Analysis of DPPH Radical Scavenging Activity

DPPH solution 0.02 mM was prepared by diluting DPPH in methanol. Then, 1 mL of tea extract was added with 1 mL of DPPH solution. After 30 minutes of dark incubation, the absorbance was read at 517 nm using spectrophotometer UV-Vis Thermoscientific GENESYS 150. The result was expressed as mg gallic acid equivalent (mg GAE/g). The standard solution is used in the form of a gallic acid solution concentrating 1 *μ*M–10 *μ*M [19].

#### 2.2.7. Analysis of Reducing Power Using the FRAP Method

The reducing power of the tea extract using the FRAP method was measured by the spectrophotometric method. FRAP reagent was made by mixing of 300 mM acetate buffer pH 3.6, 10 mM 2,4,6-tripyridyl-s-triazine (TPTZ) in 40 mM HCl and 20 mM FeCl_3_.6H_2_O with a 10: 1: 1 ratio of acetate buffer, TPTZ and FeCl_3_.6H_2_O (v/v). The reagent was then incubated at 37°C. Then, as much as 0.9 mL of reagent was reacted with 30 *μ*l of tea extract sample and 30 *μ*l of aquadest in the test tube for 4 minutes in a dark room. The absorbance was measured by a UV-Vis Spectrophotometer Thermoscientific GENESYS 150 at 593 nm [20]. The analysis uses the standard ascorbic acid standard curve (400, 420, 440, 460, and 480 *μ*M). The result was expressed as mg ascorbic acid equivalent (mg AAE/g).

#### 2.2.8. Identification of Volatile Compound

Volatile analysis used referred to the method of the previous study [21]. 5 mL tea extract was placed at a 22 mL SPME vial and extracted with DVB/CAR/PDMS 2 cm fiber at 80°C for 30 minutes. The equilibrium time was 10 minutes. Then, a 7890A GC system (Agilent Technologies, CA, USA) was combined with a 5975C XL EI/CI (Agilent Technologies) for GC-MS analysis. The chromatographic column was an HP5MS column (30 m × 0.25 mm × 0.25 *μ*m film thickness), with helium as the gas carrier, at a flow rate of 0.8 mL/min. Desorption of the volatile compound of samples was accomplished by inserting the SPME needle for 10 minutes at the injection temperature of 250°C. The MS injector was 250°C, and it was equipped with a splitless injector. The temperature column was set initially to 50°C (held for 3 min), was increased to 190°C at 5°C/min (held for 3 min), and then increased further to 220°C at 4°C/min (held for 3 min). The scan mass range was 29–550 amu. The MS Quad temperature and MS source were set at 150 and 230°C. The volatile compounds were identified by comparing their mass spectra with those stored in the National Institute of Standards and Technology (NIST14) US Government library, and LRI index was calculated based on a series of C_7_-C_23_ alkane mixtures. Quantification of volatile compounds was achieved using standard compounds allowing quantification of compounds that eluted at the same time [22].

#### 2.2.9. The Best Treatment of Tyrosinase-Treated Tea Leaf Extract

The best treatment of tyrosinase-treated tea leaf extract was determined [23]. Catechin content, the ratio of theaflavin/thearubigin, antioxidant activity, and total phenolic content were used to determine the best treatment of tyrosinase-treated tea leaf extract.

### 2.3. Statistical Analysis

Data represent the mean of triplicate analysis using ANOVA (analysis of variance) with SPSS statistical software version of 22. Differences were considered to be significant at *P* ≤ 0.05.

## 3. Result and Discussion

### 3.1. Individual Catechin and Gallic Acid Compound

The percentage of individual catechin and gallic acid of black tea treated by tyrosinase and commercial black tea could be seen in Table 1. Data showed that the percentage of gallic acid, Epicatechin (EC), Epicatechin gallate (ECG), epigallocatechin (EGC), and Epigallocatechin gallate (EGCG) was reduced during the higher concentration of tyrosinase.

**Figure 1:**
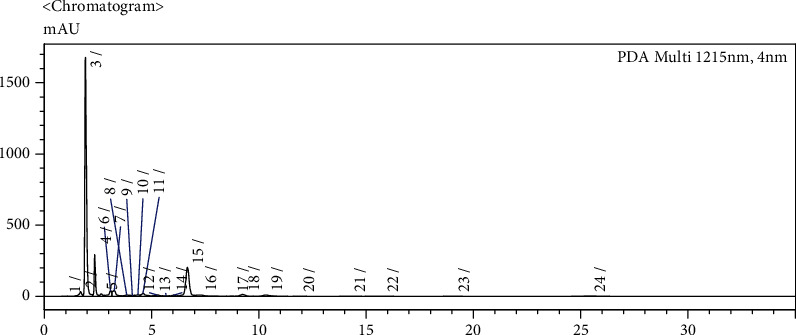
HPLC chromatogram of individual catechin and gallic acid of tyrosinase and *β*-glucosidase-treated tea leaves.

During the incubation of tea leaf extract and tyrosinase, the concentration of each catechin and gallic acid was decreased. Compared with control treatment, tea extract treated with the highest concentration of tyrosinase (1785 U/mL) had decreased the EC of 100%, 41% of ECG, 30% of EGC, and 16% of the gallic acid. As much as 90% of catechin was decreased during the processing of black tea [5]. The chromatogram of individual catechin and gallic acid of tyrosinase and *β*-glucosidase-treated tea leaves could seen in Figure 1. Incubation of tea leaf extract with tyrosinase has decreased 50% of epicatechin (EC), 75% of epigallocatechin (EGC), 60% of epicatechin gallate (ECG), and 50% of epigallocatechin gallate (EGCG) [24]. This decrease was because of the oxidation of each catechin by tyrosinase and formed theaflavins. Tyrosinase can oxidize the catechol type of catechin, i.e., epicatechin (EC), and epicatechin gallate (ECG) more than pyrogallol type catechin, i.e., epigallocatechin (EGC), and epigallocatechin gallate (EGCG) [25]. It was in line with the experiment, in which the decrease of the catechol type of catechin was more than pyrogallol type catechin because of oxidation by tyrosinase. The tyrosinase-treated tea extract has a higher content of catechin total than BOP commercial tea. It was because the clone used of tyrosinase tea extract and BOP commercial tea was different. Tyrosinase-treated tea extract was made from the superior clone in the Pagilaran Plantation, which has high quality and high productivity compared to another clone made for the BOP commercial tea [26].

### 3.2. Theaflavin and Thearubigin Content

Theaflavin and thearubigin content of tea leaf extract treated by tyrosinase and commercial black tea could be seen in Table 2. It showed that green tea treated with 1785 U/mL of tyrosinase has the highest theaflavin and thearubigin content. Besides, it has the highest ratio of TF/TR.

Theaflavin and thearubigin were not detected in the control treatment. The control treatment was the extract of fresh tea leaves after it was steamed to inactive the endogenous enzymes. Steamed tea leaves loosed the activity of the enzymes such as polyphenol oxidase and peroxidase; thus, there was no oxidation process of the catechin. Steaming can inactivated polyphenol oxidase [27] and peroxidase [28]. Data showed that the higher concentration of tyrosinase produced theaflavin and thearubigin more. This result was in line with the previous study [5], and that incubation of green tea extract with tyrosinase was three times higher than black tea. The TF/TR ratio found in black tea was divided into three categories. TF/TR ratio values up to 0.04 are included in the category of good black tea, TF/TR ratio of 0.04 to 0.08 includes the category of better quality tea, and TF/TR ratio values above 0.08 were the best tea categories [29]. Based on Table 2, the TF/TR ratio values of green tea treated with tyrosinase were in the best quality tea range, and the commercial black tea was in the better quality of black tea. Compared to BOP commercial tea, the low content of thearubigins in tyrosinase-treated tea leaf extract was because of the absence of peroxidase (POD). Black tea prepared from endogenous tea leaf enzyme, i.e., peroxidase, can further oxidize theaflavins into thearubigins and form the brown color [30]. Moreover, the oxidation process of BOP commercial tea by polyphenol oxidase and peroxidase took a long time, up to 2-4 hours (orthodox method); therefore, it could produce more content of thearubigins [31].

### 3.3. Antioxidant Activity and Total Phenolic Content

Antioxidant activity was determined by DPPH radical scavenging activity and reducing power. Antioxidant activity and total phenolic content of black tea treated by tyrosinase and commercial black tea could be seen in Table 3. It showed that the sample treated with 111 U/mL of tyrosinase was the highest antioxidant activity and total phenolic content, followed by 446 U/mL, 1785 U/mL, and commercial black tea.

According to Table 3, the control treatment has the highest content of total phenolic content. Total phenolic content was decreased during the increase of the tyrosinase concentration. Tyrosinase was a polyphenol oxidase that can be oxidized polyphenol, especially catechin [24]. Tea leaf extract has a high catechin content, as much as 30% of dry basis [5]. The higher concentration of tyrosinase provides higher catechin oxidation into phenolic content, i.e., theaflavin and thearubigin. Compared to the control treatment, the total phenolic content of tyrosinase-treated tea extract was low because it formed to theaflavin and thearubigin, which have a small concentration compared to catechin abundant in the control treatment [18]. According to the previous study [32], the total phenolic content of the black (90.79 mg GAE/g) tea was lower than green tea (117.15 mg GAE/g db).

Based on Table 3, the antioxidant activity of the tea extract decreased over time with a higher concentration of tyrosinase. A higher concentration of tyrosinase will convert catechin into theaflavin by tyrosinase, which has lower antioxidant activity. The antioxidant activity of tea leaves was reduced by tea fermentation. The tea fermentation process in producing black tea underwent the oxidation of catechin into theaflavin and thearubigin and reduced the antioxidant activity compared with green tea [33]. The antioxidant activity was in line with the total phenolic content. The highest antioxidant activity was the control treatment with the highest total phenolic content. The lowest antioxidant activity was commercial black tea (BOP), which has the lowest total phenolic content. The total phenolic content and antioxidant activity of tea have a positive correlation. The higher total phenolic content, especially catechin, showed a higher/greater antioxidant activity [34]. The order of antioxidant activity of phenolic content of the tea was EGCG (epigallocatechin gallate) > EGC (epigallocatechin) > ECG (epicatechin gallate) > theaflavin > thearubigin > EC (epicatechin) [35].

### 3.4. The Best Treatment of Tyrosinase-Treated Tea Extract

Among the treatments, tea extract that was treated with 446 U/mL of tyrosinase was the best treatment because it has great antioxidant activity, high content of catechin and total phenolic, and a high ratio of theaflavin and thearubigin. Then, the best treatment was treated with *β*-glucosidase to increase the volatile compound.

### 3.5. Volatile Compound Analysis

The volatile compounds of the tea extract treated and untreated with *β*-glucosidase could be seen in Table 4.

**Figure 2:**
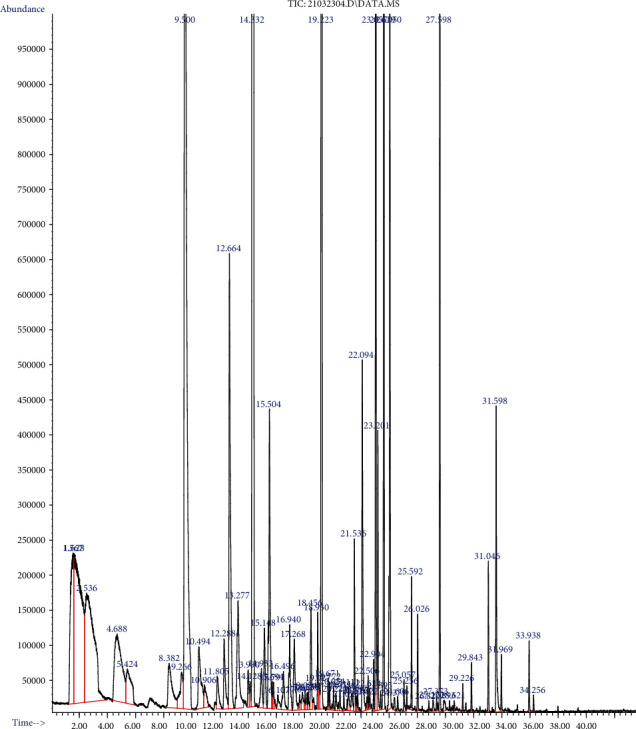
GC-MS chromatogram of the volatile compound of tyrosinase and *β*-glucosidase-treated tea leaves.

A total of 108 volatile compounds were found in the tea extract treated with *β*-glucosidase, comprised of alcohols, ketones, aldehydes, alkanes, hydrocarbons, esters, phenols, terpenes, pyrroles, pyrazine, sulfur, and others. The total of volatile compounds in the control treatment was 36.08 ppb. The tyrosinase-treated tea extract was 36.49 ppb, and the tyrosinase- and *β*-glucosidase-treated tea extract was 58.99 ppb (data not shown).  Table 4 shows an increase of volatile compounds bound glycosidically in the tea extract after being treated with *β*-glucosidase. Linalool, geraniol, linalool oxide, benzyl alcohol, methyl salicylate, and *β*-damascenone were volatile compounds that bound glycosidically with a sugar moiety [36]. Hydrolysis of the glycosidic bond of glycosides released volatile compounds and increased the aroma [37, 38]. It was found that tea leaf extract treated with tyrosinase and *β*-glucosidase has more content of volatile compounds. This was in accordance with the previous study that treated green tea extract with immobilized *β*-glucosidase, which found an extremely increasing content of volatile compounds, i.e., linalool and methyl salicylate [39]. According to Table 4, the amount of linalool, linalool oxide, *β*-damascenone and methyl salicylate found in tea leaf extract treated with tyrosinase and *β*-glucosidase was quantified of 1.923 ppb, 0.195 ppb, 0.035 ppb, and 0.957 ppb, respectively. The chromatogram of the volatile compound of tyrosinase- and *β*-glucosidase-treated tea leaves could seen in Figure 2.

It was found that the concentration of linalool, linalool oxide, and methyl salicylate found in tea leaf extract treated with tyrosinase and *β*-glucosidase increased up to 494.3%, 125.9%, and 55.,94%, respectively, compared to the control. Besides, *β*-damascenone was the new volatile compound found in the *β*-glucosidase-treated fresh tea leaf extract. These volatile compounds showed pleasing aroma such as floral (linalool) floral and sweet (*cis*-linalool oxide) [40], honey-like (*β*-damascenone) [41], and fresh and sweet (methyl salicylate) [36]. Therefore, tea leaves of PGL 15 showed that it has a good potential to produce aromatic black tea, and the application of *β*-glucosidase may produce a higher concentration of the pleasing volatile compound than untreated *β*-glucosidase tea leaf extract.

## 4. Conclusion

The application of tyrosinase in the tea extract significantly increased the theaflavin content and was in the best quality tea range. The addition of *β*-glucosidase in the tyrosinase-treated tea extract showed an increase of volatile compounds formed from glycosides. A combination of 446 U/mL of tyrosinase and 7.4 U/mL of *β*-glucosidase on the tea extract could produce a good color and pleasing aroma. The combination of these enzymes could be considered as directly processing RTD tea on the farm.

## Figures and Tables

**Table 1:** Cathecin profile and gallic acid content of tea leaf extract treated with tyrosinase.

Treatment	Tyrosinase activity (U)	Gallic acid (%)	EGC (%)	ECG (%)	EC (%)	EGCG (%)
Control		8.12 ± 0.19	13.42 ± 0.23	22.45 ± 0.08	9.42 ± 0.31	9.41 ± 0.14
Tyrosinase	111	8.19 ± 0.25	9.65 ± 0.38	12.66 ± 0.17	9.33 ± 0.13	9.37 ± 0.15
	446	7.58 ± 0.29	8.84 ± 0.34	9.85 ± 0.2	9.04 ± 0.19	9.91 ± 0.19
	1785	6.82 ± 0.27	9.35 ± 0.26	13.21 ± 0.09	0.00	9.28 ± 0.03
Commercial black tea (BOP)		11.05 ± 0.3	7.35 ± 0.31	7.56 ± 0.3	7.54 ± 0.1	7.84 ± 0.14

Values are expressed as mean ± standard deviation of three replications.

**Table 2:** Theaflavin and thearubigin content of tea leaf extract treated with tyrosinase.

Treatment	Tyrosinase activity (U)	TF (%)	TR (%)	TF/TR
Control		0^a^	0^a^	0
Tyrosinase	111	3.07 ± 0.10^c^	2.69 ± 0.18^b^	1.15
	446	3.54 ± 0.02^d^	2.53 ± 0.91^b^	1.55
	1785	5.97 ± 0.09^e^	3.56 ± 0.32^c^	1.69
Commercial black tea (BOP)		1.3 ± 0.01^b^	21.45 ± 0.31^d^	0.06

Values are expressed as mean ± standard deviation of three replication. Different letters ^a,b,c^ in the same column indicate significant differences (*P* < 0.05) between samples.

**Table 3:** Antioxidant activity and total phenolic content of tea leaf extract treated with tyrosinase.

Treatment	Tyrosinase activity (U/mL)	Total phenolic content (mg GAE/g db)	DPPH radical scavenging activity (mg GAE/g db)	Reducing power (mg AAE/g db)
Control		148.10 ± 5.36^d^	103.03 ± 3.69^e^	188.69 ± 2.37^d^
Tyrosinase	111	139.87 ± 7.68^c^	93.48 ± 2.88^d^	157.87 ± 9.53^c^
	446	131.92 ± 5.70^c^	88.28 ± 2.3^c^	138.53 ± 5.68^b^
	1785	117.72 ± 4.89^b^	78.63 ± 1.87^b^	113.24 ± 5.02^a^
Commercial black tea (BOP)		64.08 ± 3.09^a^	37.85 ± 0.80^a^	105.48 ± 1.77^a^

Values are expressed as mean ± standard deviation of three replication. Different letters ^a,b,c^ in the same column indicate significant differences (*P* < 0.05) between samples.

**Table 4:** The volatile compound of the treated and untreated tea extract with *β*-glucosidase.

Compound	CAS	RI^∗^	RI^∗∗^	LRI	Concentration (ppb)		
					Control	Tyrosinase	Tyrosinase-*β*-glucosidase
Linalool	78-70-6	1099	1101	1104	0.389 ± 0.027	0.138 ± 0.024	1.923 ± 0.186
*cis*-Linalool oxide	5989-33-3	1069	1078	1076	0.218 ± 0.02	0.085 ± 0.009	0.194 ± 0.02
Linalool oxide	14049-11-7	1071	1072	1179	0.155 ± 0.02	0.100 ± 0.017	0.195 ± 0.023
*β*-Damascenone	23726-93-4	1386	1385	1388	—	—	0.035 ± 0.011
Methyl salicylate	119-36-8	1193	1235	1266	0.173 ± 0.021	0.074 ± 0.027	0.957 ± 0.033

Values are expressed as mean ± standard deviation of three replication. ^∗^RI: RI of references using the DB-5MS column [16]; ^∗∗^RI: RI of references using the HP-5MS column [22]; LRI: retention indices calculated in the experiment.

## Data Availability

The data used to support the findings of this study are included within the article.
